# Impact of molecular testing in advanced melanoma on outcomes in a tertiary cancer center and as reported in a publicly available database

**DOI:** 10.1002/cnr2.1380

**Published:** 2021-06-09

**Authors:** Maya Dimitrova, Min Jae Kim, Iman Osman, George Jour

**Affiliations:** ^1^ Langone Medical Center New York University New York USA; ^2^ School of Medicine New York University New York USA

**Keywords:** cancer genetics, melanoma, mutations, targeted therapy

## Abstract

**Background:**

In patients with advanced melanoma (MM), genomic profiling may guide treatment decisions in the frontline setting and beyond as specific tumor mutations can be treated with targeted therapy (TT). The range of panel sizes used to identify targetable mutations (TM) can range from a few dozen to whole exome sequencing (WES).

**Aim:**

We investigated the impact of panel size and mutation status on first‐line treatment selection and outcomes in MM.

**Methods and Results:**

We analyzed data for 1109 MM patients from three cohorts: 169 patients at NYULH and profiled with the 50 gene Ion Torrent panel (IT), 195 patients at MSKCC, profiled with the 400‐gene MSK‐IMPACT panel (MSK‐I) and 745 patients at seven different sites profiled with WES. Data for cohorts 2 and 3 were extrapolated from the publicly available cBioPortal.

Treatment information was available for 100%, 25%, and 0% of patients in cohort 1, 2, and 3, respectively. BRAF and NRAS were among the top five most commonly mutated genes in the IT and MSK‐I, whereas for WES only BRAF was a top five mutation. There was no significant difference in OS for BRAF MUT patients treated with immune checkpoint inhibitors (ICI) vs TT in cohort 1 (*P* = .19), nor for BRAF MUT patients from cohort 1 treated with ICI vs those from cohort 2 treated with TT (*P* = .762).

**Conclusion:**

Public datasets provide population‐level data; however, the heterogeneity of reported clinical information limits their value and calls for data standardization. Without evidence of clear clinical benefit of a larger panel size, there is a rationale for adopting smaller, more cost effective panels in MM.

## 1 INTRODUCTION

Activating somatic mutations in *BRAF* are present in approximately 50% of advanced melanomas. Molecular testing for this mutation has become a standard of care as recommended by the National Comprehensive Cancer Network (NCCN) and the European Society for Medical Oncology (ESMO) for stage III or stage IV disease.^1^, ^2^ The combination of *BRAF* and MEK targeted inhibition (TT) led to the first significant improvements in progression free survival of patients with metastatic melanoma.^3^, ^4^ Targeted therapy and checkpoint inhibitors are both preferred front line treatments for metastatic melanoma.^2^ However, at this time, there are no formal recommendations on how these therapies should be sequenced in patients with *BRAF* positive tumors. In addition, depending on the clinical practice, the panels used to identify targetable alterations can range from small panels of 30 to 40 genes to whole exome sequencing (WES).^5^ Next generation sequencing (NGS) and WES have demonstrated utility in identifying other potentially clinically relevant mutations in melanoma but none that change frontline treatment at this time.^6^, ^7^, ^8^ To our knowledge, there are no studies investigating the size of a molecular panel and its impact on the choice of frontline treatment in cutaneous metastatic melanoma. Although WES may identify potential biomarkers for response to immunotherapy such as microsatellite instability (MS), homologous recombination scores, or tumor mutational burden (TMB), none has been validated in prospective trials in a variety of solid tumors including melanomas.^9^

Herein, we examine the utilization of a targeted molecular sequence panel in a cohort of patients with advanced melanoma at NYULH and compare that with publicly available datasets from other tertiary medical centers using larger panels ranging from 400+ genes to WES to examine the effects that molecular panel sizes and mutational information have on treatment selection and patient outcomes.

## 2 PATIENTS AND METHODS

### 2.1 Patient selection

We analyzed data for 1109 MM patients from three cohorts. Cohort 1 included 169 patients with advanced (stage III or V) melanoma enrolled at NYULH (Cohort 1) and profiled with the 52 gene Ion Torrent panel (IT). Cohort 2 included 195 patients enrolled at MSKCC (Cohort 2), profiled with the 400‐gene MSK‐IMPACT panel (MSK‐I). Cohort 3 included 745 patients enrolled at seven different sites reporting information to cBioPortal and profiled with WES.

### 2.2 Data collection

Data for cohorts 2 and 3 were extrapolated from publicly available data using cBioPortal (completion of data acquisition October 20, 2019). We examined sex, age, cancer stage, lactate dehydrogenase levels (LDH), Eastern Cooperative Oncology Group (ECOG) performance status, number of metastatic sites, *BRAF* status, treatment received and response to treatment as available by the reported data. We searched clinicaltrials.gov for actively recruiting clinical trials as of April 10, 2020 on patients with advanced melanoma harboring each of the top 50 mutations (excluding *BRAF*) as identified by the NYULH, MSK‐IMPACT, and WES panels.

### 2.3 Statistical analysis

We tested associations between molecular data, treatment choice and overall survival (OS), adjusting for baseline characteristics when available. Statistical tests including t tests, log rank test for survival analysis were carried using Graphpad Prism V.8 (*P* < .05).

## 3 RESULTS

### 3.1 Clinicopathological characteristics of the three cohorts studied

The NYULH cohort of patients consisted of 169 patients with a mean age of diagnosis of 61.6 (Table 1). The male to female ratio was 1:1. 40% of the patients had molecular testing when presenting with stage III disease and 60% were stage IV. The majority of patients (64%) had a normal LDH and an excellent performance status of 0 (60%). In cohort 2 (*n* = 195), all data points were missing with the exception of gender distribution (Male = 115, Female = 80). It was not possible to know the mean age of the patients in MSK‐IMPACT cohort though the M:F ratio was similar. Stage, LDH, and ECOG status were unknown. Although there is more information available in aggregate for the rest of the cBioPortal cohort (Cohort 3), the absolute percentages for stage, LDH, and ECOG are low (<10% of total with concrete data).

**TABLE 1:** Summary of the clinical characteristic of the three cohorts

Characteristic		NYU *N* = 169	MSK‐IMPACT *N* = 195	Other^a^ *N* = 745
Age at diagnosis (y)	Mean	61.6	N/A	68.2
	Unknown—no. (%)	0	195 (100)	103 (13.8)
Age at molecular testing (y)	Mean	65.3	N/A	52
	Unknown—no. (%)	0	195 (100)	626 (84%)
Gender—no. (%)	Male	100 (59)	115 (59)	422 (56.6)
	Female	69 (41)	80 (41)	257 (34.5)
	Unknown	N/A	N/A	66 (8.9)
Stage at molecular testing—no. (%)	Stage III	68 (40)	N/A	12 (1.6)
	Stage IV	101 (60)	N/A	164 (22)
	Unknown	N/A	195 (100)	569 (76.4)
Number of metastatic sites—*N* (range)		169 (0‐6)	N/A (N/A)	176 (1‐7)
LDH—no. (%)	Normal	108 (64)	N/A	58 (7.8)
	High	30 (18)	N/A	48 (6.4)
	Unknown	31 (18)	195 (100)	639 (85.8)
ECOG performance status score—no. (%)	0	101 (60)	N/A	29 (3.9)
	≥1	50 (30)	N/A	37 (5)
	Unknown	18 (10)	195 (100)	679 (91.1)

*Note*: Given the lack of data for MSK‐IMPACT cohort (cohort #2), clinical and demographical information are presented in the text.

Abbreviation: N/A, not applicable.

^a^ Includes: Broad, DFCI, Vanderbilt, TGCA—All had testing done using WES.

### 3.2 Comparison of targeted panels and their genomic design reveals significant heterogeneity

**FIGURE 1:**
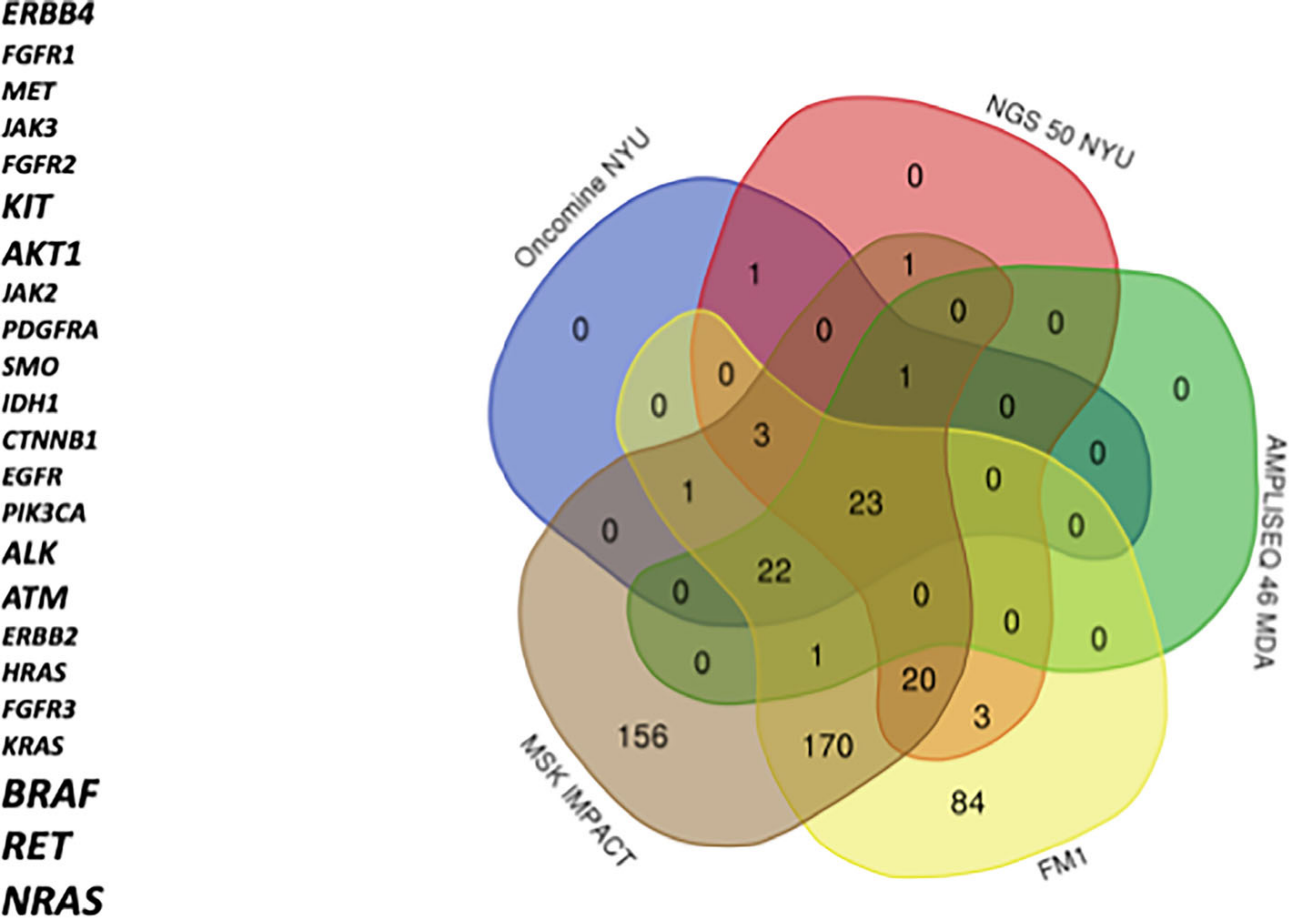
Twenty‐three genes in common across targeted platforms

We looked at shared genes in the panel design across the targeted molecular panels used by different institutions (NYULH, MD Anderson, MSKCC, and Foundation Medicine) and identified 23 genes that overlapped across five panels (Figure 1). We noticed a significant heterogeneity in the structure of these “targeted panels.” Some panels included more than 300 genes. Others were more restrictive, focusing on oncogenes and tumor suppressor genes with an FDA indication, such as the NYULH Oncomine panel. When focusing on the top five most commonly mutated genes in the cohorts, we found that the five most common alterations between the three are also very heterogeneous and noted that only *BRAF* appears among all three. For instance, while *BRAF* and NRAS appear as expected among the most commonly mutated genes in cohorts 1 and 2, *NRAS* does not appear as a top five mutation in the WES panels. Instead, the other four most common gene alterations in this group *are LRP1B, PCLO, FAT4*, and *MGAM*. Interestingly, *BRAF* was not the most common mutation in the melanoma samples tested by the IMPACT panel but the *TERT* hot spot promoter mutation appeared with a frequency of 73%.

### 3.3 Impact of upfront molecular testing on treatment decisions and outcomes in NYULH cohort

Frontline treatment modality was only available for the NYULH cohort. Based on the data reported on cBioPortal, we were able to infer that 25% of the patients in the MSK‐IMPACT cohort received TT by analyzing the referenced trial on the portal (Table 2). We observed that regardless of mutation status (*BRAF* mutant vs *BRAF* wild type vs other) immunotherapy was the first‐line treatment choice in Cohort 1 (Table 3). Targeted therapy was more commonly chosen over immunotherapy as a second line treatment in *BRAF* mutant melanoma. At the time of data review, 67.5% of patients in Cohort I were still alive, 30.2% were deceased and there was insufficient data on 2.4% regarding survival. This compared similarly to the numbers that were available for Cohort 2 with 61%, 39%, and 0%, respectively, however, the numbers for Cohort 3 were skewed by gaps in the reported data. In cohort 1, 36% (16/45) of *BRAF* MUT patients received first‐line TT. There was no significant difference for *BRAF* MUT patients treated with ICI vs TT in cohort 1 in OS (*P* = .19), nor for *BRAF* MUT patients from cohort 1 treated with ICI vs those from cohort 2 treated with TT (OS *P* = .762). There was no data on sequence of treatment in the studies that were reported in cohort 3 or on the response to such treatment.

**TABLE 2:** Clinical outcomes for the three cohorts

Outcome		NYU *N* = 169	MSK‐IMPACT *N* = 195	Other^a^ *N* = 745
Treatment received—no. (%)	Immunotherapy	134 (79.3)	Unknown	32 (4.3)
	Targeted	31 (18.3)	Unknown	66 (8.9)
	None	26 (15.4)	Unknown	Unknown
	Unknown	N/A	195 (100)	679 (91.1)
First‐line treatment after molecular testing—no. (%)	Immunotherapy	131 (77.5)	Unknown	Unknown
	Targeted	16 (9.5)	Unknown	Unknown
	None	26 (15.4)	Unknown	Unknown
	Unknown	N/A	195 (100)	745 (100)
BRAF mutation status—no. (%)	V600E/K	45 (26.6)	N/A	N/A
	Other	11 (6.5)	N/A	N/A
	Wild type	113 (66.9)	114 (58.5)	323 (43.3)
	Unclassified	N/A	81 (41.5)	414 (55.6)
	Unknown	N/A	N/A	8 (1.1)
Alive status—no. (%)	Alive	114 (67.5)	139 (71.3)	272 (36.5)
	Dead	51 (30.2)	56 (28.7)	345 (46.3)
	Unknown	4 (2.4)	N/A	128 (17.2)
Median follow up from molecular testing—mos. (range)		24 (0‐52)	Unknown	Unknown

Abbreviation: N/A, not applicable.

^a^ Includes: Broad, DFCI, Vanderbilt, TGCA. Testing done with WES.

**TABLE 3:** Treatment based on mutation status for cohort 1

Mutation	Line of treatment	Treatment received (% of total)
BRAF	First	ICI (74), TT (21)
	Second	ICI (44), TT (48)
	Third	ICI (36), TT (45)
NRAS	First	ICI (97), TT (3)
	Second	ICI (85), TT (14)
	Third	N/A
TP53	First	ICI (90), Other (10)
	Second	ICI (100)
	Third	ICI (100)
APC	First	ICI (100)
	Second	
	Third	
CDKN2A	First	ICI (100)
	Second	ICI (100)
	Third	N/A
Other	First	ICI (100)
	Second	
	Third	
None	First	ICI (100)
	Second	ICI (85), TT (14)
	Third	ICI (100)

Abbreviations: ICI, immunecheckpoint inhibitor; TT, targeted therapy.

### 3.4 Comparison of genomic events detected between cohorts 1, 2, and 3 and their impact on eligibility for clinical trials

**FIGURE 2:**
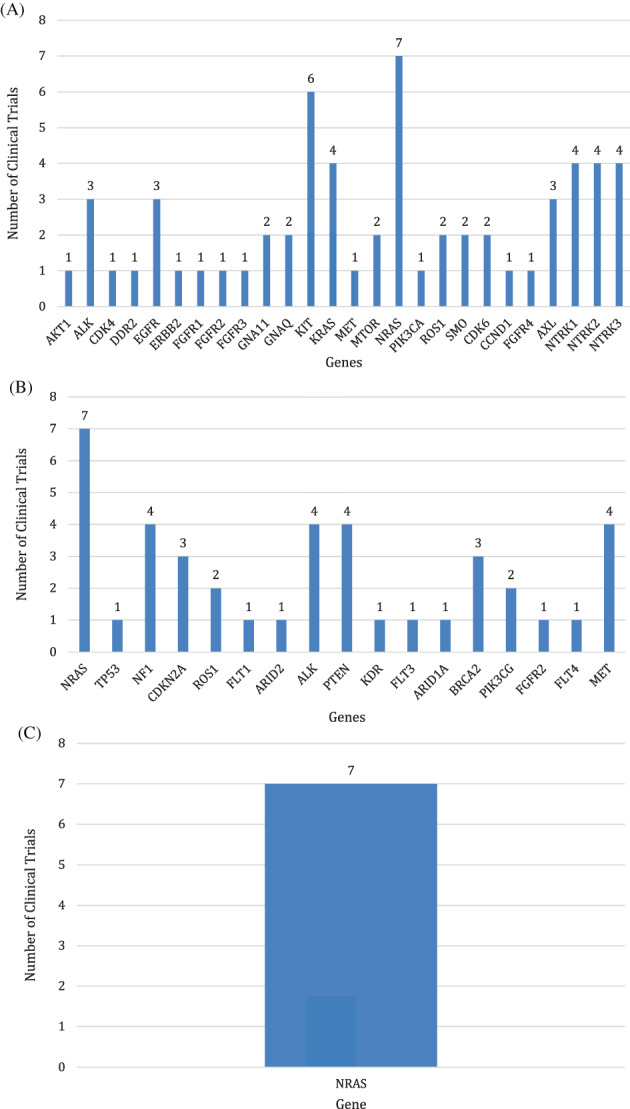
Number of eligible clinical trials out of the 50 most frequently occurring mutations in cohorts 1 to 3. A, A total of 26 targets detected in Cohort 1 for which there was an enrolling clinical trial. B, A total of 17 targets detected in Cohort 2 for which there was an enrolling clinical trial. C, A total of 1 target detected in Cohort 3 for which there were enrolling clinical trials

We then compared the top 50 most frequently affected genes from the three genomic platforms (NYULH, MSK‐IMPACT, and WES) and their impact on trial inclusion. Based on the NYULH panel, genomic information from these genes would have served as an inclusion criteria in 61 actively recruiting clinical trials, whereas this number decreases to 39 trials using the top 50 most frequent genes from the MSK‐IMPACT panel and is only seven trials (for one candidate gene‐NRAS only) using WES (Figure 2 and Table S1).

### 3.5 Comparison of the database parameters used to source cohorts 1, 2, and 3

There was significant heterogeneity in the data reported by each of the trials that was included in the cBioPortal database and from which the molecular data was derived. The lack of standardized reporting of clinical information made it challenging to compare across trials and datasets. For example, sequencing of treatment types was not identifiable using the cBioPortal portal. That is, if treatment was reported at all.

## 4 DISCUSSION

Large panels and WES can identify many more mutations than guidelines recommend testing for or for which there are available treatments.^10^ Small molecular panels that fulfill international guidelines and FDA recommended therapeutics may be the most cost efficient and easily applicable paradigms to use in the frontline setting. In fact, at this time, there is no data from randomized prospective trials to dictate sequence of therapy in advanced and metastatic melanoma based on molecular profiling; current recommendations are made based on retrospective reviews.^11^, ^12^ As demonstrated by cohort I, there does not appear to be a significant difference in outcome whether patients received IO or TT first. One retrospective review suggests that IO followed by TT leads to superior responses compared to the inverse.^13^ Other studies have not found a difference in survival based on sequence but suggest there might be a higher rate of response to IO when it is given after TT.^14^, ^15^ In a cohort of Italian patients, those who received ipilimumab prior to vemurafenib or dabrafenib had better outcomes compared to patients who were treated with TT first.^16^ In general, most clinicians are starting treatment with immunotherapy unless a patient has large volume, symptomatic disease. TT has been shown to be superior in this clinical scenario.^17^ Given this area of clinical uncertainty, there are two ongoing clinical trials that may establish the sequence of IO and TT: the SECOMBIT (NCT02631447) and DREAMseq studies (NCT02224781).

There is evidence that larger testing platforms may provide more data to guide clinical decision making. In fact, a retrospective analysis of 10 000 patients profiled using the MSK‐IMPACT panel demonstrated that patients' whose tumors had a high TMB had better outcomes with IO vs non‐IO treatments.^9^ While this finding may be relevant to justify IO regimens in numerous solid tumor types, its usefulness in the particular setting of melanoma remains limited. Although melanoma has already been identified as having the highest TMB from the initial TCGA study, TMB is not used to stratify patients to IO vs other therapies. These findings further support the argument that some of the genomic data generated by large panels is important in addressing research question, but remains of limited use in the current clinical setting.^18^

**FIGURE 3:**
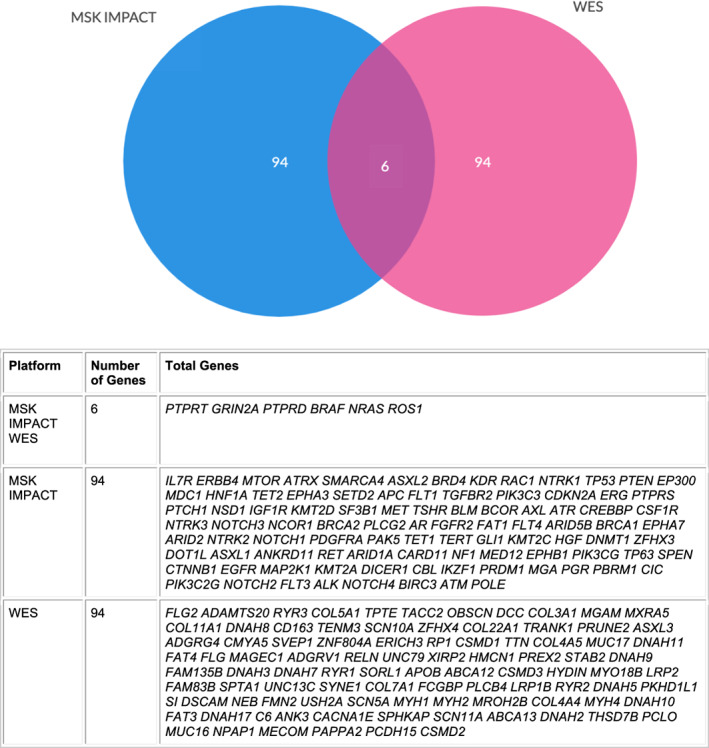
Top 100 Genes in WES and MSKIMPACT overlap

What may be more clinically relevant for patients is the identification of molecular targets for which there are clinical trials and therefore further therapeutic options. Focusing on the 50 most frequent genes between the larger molecular panels used in cohorts 2 and 3, there are only six in common between the two panels (*PTPRT, GRIN2A, PTPRD, BRAF, NRAS, ROS1*) (Figure 3). *ROS1* is the only gene from this set that was not identified by our own IT panel for which there are two clinical trials currently open (NCT02568267 and NCT02465060). Twenty nine of the 50 genes (excluding *BRAF*) in the IT panel were associated with clinical trials with 61 active trials at the time of this publication vs 25 genes of the top 50 in MSK‐IMPACT (*P* < .0001) out of a total 400+ genes. This again demonstrates the utility of a limited gene panel in identifying a significant number of eligible clinical trials once a patient has progressed on standard therapy. In fact, of the top 50 mutations identified by WES, only one was associated with clinical trial eligibility (Figure 2 and Table S1).

There are several limitations to our study. Our sample size in cohort I was the smallest of the three cohorts and the sample sizes across the different cohorts varied significantly. Our cohort also included a significantly larger proportion of stage III patients, which lowered the proportion of *BRAF* mutated patients to less than 50%. However, our 169 patients compare favorably with the sample sizes of the individual studies that have reported into cBioPortal and we think this population is comparable to what is seen at other tertiary centers. There was a lack of reported patient data in Cohorts 2 and 3, as results were pooled from larger numbers of trials with no standardized data sets, which limited our ability to compare across cohorts. Finally, this was a retrospective review of one institutional experience, which makes our data susceptible to bias. However, our results support other published studies to date which demonstrate that sequencing immunotherapy as a frontline therapy may confer the best survival advantage to patients, or at least be noninferior to targeted therapy.^11^, ^13^, ^14^, ^15^, ^16^, ^17^

In conclusion, we have demonstrated that using a small, targeted panel of only 50 genes provides sufficient information to guide clinical decision making in the frontline treatment of advanced or metastatic melanoma. This is very important given the financial constraints associated with lack of reimbursements by Centers for Medicare and Medicaid Services (CMS) and third party payers when using larger panels. Although large panels and WES may provide actionable information in relapsed or refractory patients, their cost does not seem justified in the initial setting. Thus, we propose reserving these panels for progressive and/or refractory disease. Our study also highlights the challenges in using publicly available data sets to answer clinical questions. These databases are plagued by the heterogeneity of the different data fields collected. Thus, the utility of the “meta‐data” collected is severely hampered without a standardized format for reporting patient level data in the public domain. To that end, we propose the adoption of the format we use in our institutional interdisciplinary melanoma cooperative group (IMCG) database to overcome this shortage and harmonize data fields across publicly available databases (Table S2).

## CONFLICT OF INTEREST

The authors declare no conflicts of interest.

## AUTHORS' CONTRIBUTIONS

All authors had full access to the data in the study and take responsibility for the integrity of the data and the accuracy of the data analysis. *Conceptualization*, I.O. and G.J.; *Methodology*, I.O. and G.J.; *Validation*, M.D., M.J.K., I.O., and G.J.; *Investigation*, M.D., M.J.K., and G.J.; *Formal Analysis*, M.D. and G.J.; *Data Curation*, M.J.K.; *Writing ‐ Original Draft*, M.D. and G.J.; *Writing ‐ Review & Editing*, M.D. and G.J.; *Visualization*, M.D., M.J.K., I.O., and G.J.; *Supervision*, I.O. and G.J.; *Project Administration*, I.O. and G.J.; *Funding Acquisition*, I.O.

## ETHICAL STATEMENT

All patients were accrued to the IRB‐approved New York University Interdisciplinary Melanoma Cooperative Group (NYU IMCG) protocol with patient consent.

## Supporting information

**Table S1**. Actively enrolling clinical trials based on eligible mutations.
**Table S2**. Database parameters.

## Data Availability

The data that support the findings of this study come from two sources: one part of the data is openly available in cBioPortal at cbioportal.org. The other part of the data that support the findings of this study are available on request from the corresponding author. The data are not publicly available due to privacy or ethical restrictions.
