# Supplementary material for: Impact of molecular testing in advanced melanoma on outcomes in a tertiary cancer center and as reported in a publicly available database
Source: Cancer Rep (Hoboken). 2021 Jun 9;4(4):e1380. doi: 10.1002/cnr2.1380 (PMC8388178; doi:10.1002/cnr2.1380)
Supplement: Supplementary file 1 — Table S1. Actively enrolling clinical trials based on eligible mutations. Table S2. Database parameters. [file CNR2-4-e1380-s001.pdf]

Table S1. Actively Enrolling Clinical Trials Based on Eligible Mutations

NYU-IT	NCT Designation	Number of Trials	MSK-Impact	NCT Designation	Number of Trials	WES	NCT Designation	Number of Trials
AKT1	NCT03190174, NCT02693535	1	TERT		0	TTN		0
ALK	NCT02091141, NCT02568267, NCT02465060	3	PTPRT		0	MUC16		0
ATM		0	NRAS	NCT02465060, NCT02729298, NCT02974725, NCT04059224, NCT04109456, NCT03932253, NCT03973151	7	DNAH5		0
CDK4	NCT02693535	1	PAK5		0	LRP1B		0
CTNNB1		0	GRIN2A		0	PCLO		0
DDR2	NCT02465060	1	TP53	NCT02783300	1	ADGRV1		0
EGFR	NCT02729298, NCT02465060, NCT03841110	3	NF1	NCT02465060, NCT03634982	2	ANK3		0
ERBB2	NCT02465060	1	PTPRD		0	FAT4		0
ERBB3		0	CDKN2A	NCT02693535	1	CSMD2		0
ERBB4		0	TP63		0	PKHD1L1		0
ESR1		0	ERBB4		0	CSMD1		0
FGFR1	NCT02465060	1	ROS1	NCT02465060, NCT02568267	2	USH2A		0
FGFR2	NCT02465060	1	EPHA7		0	DNAH7		0
FGFR3	NCT02465060	1	FAT1		0	DNAH8		0
GNA11	NCT02465060, NCT03947385	2	KMT2A		0	CSMD3		0
GNAQ	NCT02465060, NCT03947385	2	KMT2C		0	RP1		0
HRAS		0	KMT2D		0	APOB		0
IDH1		0	PIK3C2G		0	MGAM		0
IDH2		0	FLT1	NCT02693535	1	HYDIN		0
JAK1		0	NOTCH3		0	MUC17		0
JAK2		0	NTRK3		0	DNAH9		0
JAK3		0	NOTCH4		0	FLG		0
KIT	NCT02465060, NCT02501551, NCT01738139, NCT03374839, NCT02571036, NCT03767348	6	ARID2	NCT03925350	1	SYNE1		0
KRAS	NCT02974725, NCT02729298, NCT03989115, NCT03634982	4	ALK	NCT02091141, NCT02568267, NCT02465060	4	XIRP2		0
MET	NCT02465060	1	PTEN	NCT03131908, NCT02583542, NCT03190174, NCT03925350	4	PCDH15		0
MTOR	NCT02465060, NCT03190174	2	APC		0	DNAH3		0

NRAS	NCT02465060, NCT02729298, NCT02974725, NCT04059224, NCT04109456, NCT03932253, NCT03973151	7	KDR	NCT03329950, NCT02693535	2	MXRA5		0
PDGFRA		0	IL7R		0	THSD7B		0
PIK3CA	NCT02465060	1	MGA		0	DSCAM		0
RAF1		0	PGR		0	SPHKAP		0
RET		0	CARD11		0	ZFHX4		0
ROS1	NCT02465060, NCT02568267	2	EPHA3		0	NRAS	NCT02465060, NCT02729298, NCT02974725, NCT04059224, NCT04109456, NCT03932253, NCT03973151	7
SMO	NCT02465060, NCT04007744	2	ZFHX3		0	RYR1		0
CDK6	NCT02465060, NCT02693535	2	FLT3	NCT03329950, NCT02693535	2	SCN10A		0
CCND1	NCT02465060	1	ARID1A	NCT03925350	1	NEB		0
FGFR4	NCT02465060	1	BRCA2	NCT04187833, NCT02693535, NCT03925350	3	FAT3		0
MYC		0	IKZF1		0	ERICH3		0
MYCN		0	CTNNB1		0	DNAH10		0
ABL1		0	PIK3CG	NCT02583542, NCT03190174	2	PTPRT		0
AKT3		0	PLCG2		0	DCC		0
AXL	NCT02729298, NCT03425279, NCT02988817	3	BRD4		0	GRIN2A		0
ERG		0	EPHB1		0	LRP2		0
ETV1		0	ANKRD11		0	PREX2		0
ETV4		0	FGFR2	NCT02465060	1	UNC13C		0
ETV5		0	FLT4		0	COL4A4		0
NTRK1	NCT02465060, NCT03708328, NCT02568267, NCT02576431	4	MET	NCT02465060	1	RELN		0
NTRK2	NCT02465060, NCT03708328, NCT02568267, NCT02576431	4	ATRX		0	SCN11A		0
NTRK3	NCT02465060, NCT03708328, NCT02568267, NCT02576431	4	SPEN		0	RYR2		0
PPARG		0	TET2		0	MROH2B		0
AR		0	GLI1		0	ADGRG4		0

Table S2. Database Parameters

NYU	cBioPortal	Standardized Data Set
Stage	Stage	Stage
Date of Initial Diagnosis	Age at Diagnosis	Age at Diagnosis
LDH	LDH	LDH
Primary Site	Primary Site	Primary Site
Sex	Sex	Sex
ECOG		ECOG
Brain Mets	Metastatic Site	Metastatic Sites
Age at Molecular Testing	Age at Procurement	Age at Molecular Testing
Stage at Molecular Testing		Stage at Molecular Testing
Date of Molecular Testing		Tissue Source Site
Specimen for Molecular Testing	Tissue Source Site	Mutation Status
Mutation Status	Mutation Status	1st Line Treatment
1st Line Treatment	Treatment	Duration of Response to 1st Line Treatment
Duration of 1st Line Treatment		2nd Line Treatment
1st Line Treatment Best Response		Duration of Response to 2nd Line Treatment
2nd Line Treatment		3rd Line Treatment
2nd Line Treatment Best Response		Duration of Response to 3rd Line Treatment
3rd Line Treatment		Overall Survival
Duration of 3rd Line Treatment		Length of Follow Up
3rd Line Treatment Best Response		Survival at Last Follow Up
Date of Last Follow Up		
Survival at Last Follow Up		
Overall Survival	Overall Survival	
Disease Free Status	Progression Free Status	
